# Development of new colloid osmotic pressure measurement method using ultrafiltration membrane during cardiopulmonary bypass

**DOI:** 10.1051/ject/2023028

**Published:** 2023-12-15

**Authors:** Takeshi Matsumoto, Kiyoshi Yoshida, Tomotaka Shinohara, Eiji Miyoshi, Takayoshi Ueno

**Affiliations:** 1 Department of Functional Diagnostic Science, Division of Health Sciences, Osaka University Graduate School of Medicine 1–7, Suita-shi Osaka-fu 565-0871 Japan; 2 Department of Nursing Practice Development, Division of Nursing Science, Osaka University Graduate School of Medicine 1–7, Suita-shi Osaka-fu 565-0871 Japan; 3 Department of Future Medical therapy, Division of Health Sciences, Osaka University, Graduate School of Medicine 1–7, Suita-shi Osaka-fu 565-0871 Japan

**Keywords:** Ultrafiltration, Filter, Hemoconcentrator, Edema

## Abstract

*Background*: Clinical practice of measuring colloid osmotic pressure (COP) was abandoned after correcting hypoosmolarity did not improve overall patient outcomes. However, the use of albumin and colloidal solutions has contributed to maintaining intraoperative and postoperative fluid balance at lower levels. Reduced perioperative fluid balance is consistently reported to have positive effects on clinical outcomes. Priming solutions for cardiopulmonary bypass typically include colloids; however, the optimal type of priming solution has not yet been determined. Stricter COP management may further improve postoperative courses. To achieve this, the widespread adoption of a measurement method suitable for COP monitoring during cardiopulmonary bypass is required. *Methods*: A test circuit was made which measured COP using an ultrafiltration membrane method based on the changes in hydrostatic pressure that occurs across a semipermeable membrane. We then compared the measurements obtained using this method with colloidal osmometer measurements. *Results*: COP measurements were obtained for a total of 100 tests (10 times each for 10 test solutions). The evaluation parameters included simultaneous reproducibility, correlation with the colloid osmometer, and measurement time. The results demonstrated high accuracy of the ultrafiltration membrane method, simultaneous reproducibility within 3%, a high positive correlation with the colloid osmometer (correlation coefficient: *R*^2^ = 0.99; *p* < 0.01), and equal time required for measurement. *Conclusion*: Measuring COP using ultrafiltration membranes solves problems within existing measurement methods. Although further improvements in the method are necessary, it has implications for future research and clinical applications.

## Introduction

Colloid osmotic pressure (COP) is generated primarily by serum albumin in the human body. Cardiopulmonary bypass used during open-heart surgeries lowers COP due to crystalloid hemodilution [1]. The clinical practice of measuring COP was abandoned, as correcting for hypoosmolarity did not improve patient outcomes [2–5]. The use of albumin and colloid solutions in heart surgery did not affect the risk of postoperative adverse events compared with patients for whom crystalloids were used. However, the use of albumin and colloidal solutions has contributed to maintaining perioperative and postoperative fluid balance at lower levels [6, 7]. Reduced perioperative fluid balance is consistently reported to have positive effects on clinical outcomes (clinical outcome score, duration of hospital stay, and increase in body weight) [8–10]. In case we expect these secondary effects, the use of albumin and colloids is supported.

During cardiopulmonary bypass, COP decreases when the priming solution does not contain colloids [9]. Therefore, priming solutions typically contain colloids. However, the optimal type of priming solution has not yet been determined. Similar to the selection of a substitution fluid for blood loss during cardiopulmonary bypass, no clear parameters exist for selecting a colloid.

COP is correlated with fluid balance and could serve as a reliable parameter for colloid use. Low COP increases edema, hemorrhage volume, duration of low output syndrome, and duration of ICU stay [11–14]; in contrast, high COP causes hypervolemia, which can exert adverse effects on hemodynamics. In addition, hypertonic colloidal osmotic fluids have been implicated as a risk factor for acute kidney injury [15–17]. However, a small to moderate volume of colloid poses little risk of adverse events, and COP monitoring may not be mandatory. However, this alone is insufficient grounds to overlook the importance of COP measurement. Stricter control of perioperative COP may further improve postoperative courses.

At present, because COP measurements are difficult to obtain, they are rarely taken. Colloid osmometers are required to measure COP, but they are expensive and not widely used. One possible substitute method is the calculation of COP from the albumin concentration [18]. However, the disadvantages of this method are low accuracy, an error rate of about 10%, and a considerable time requirement [19]; moreover, synthetic colloids, such as hydroxyethyl starch, are not reflected in the calculation. Therefore, there is currently no established COP monitoring method suitable for use during cardiopulmonary bypass.

In this study, we devised a simple method for measuring COP during cardiopulmonary bypass. The ultrafiltration membrane used during cardiopulmonary bypass has a semipermeable membrane structure. Hence, our proposal is to directly and easily measure COP using ultrafiltration membranes. Accordingly, a test circuit was devised, and its usefulness was examined by comparing COP values measured using the ultrafiltration membrane method with those obtained through the colloid osmometer method.

## Materials and methods

### Ethical approval

This study does not involve human and/or animal subjects; therefore, ethical approval was not required.

### COP measurement principle

COP is equivalent to the hydrostatic pressure resulting from the differing concentrations of substances unable to pass across a semipermeable membrane between solutions. Only the concentration difference of the substances unable to cross the semipermeable membrane has an effect, not the vessel size or the relative amounts of solution. An ultrafiltration membrane is a container in which a semipermeable membrane separates blood on one side from the filtrate on the other side. Under normal usage of an ultrafiltration membrane, the inside of the hollow fiber is filled with blood, whereas the outside of the hollow fiber (the filtrate side) is filled with crystalloid (filtrate) exuded from blood by ultrafiltration. COP is measured with the inside and outside of the membrane filled with blood and filtrate, respectively; aligning the levels of both fluids to eliminate any hydrostatic pressure difference; and then measuring the static hydrostatic pressure difference that occurs at standby time. Standby time was defined as the time spent waiting for the solvent to be transferred by COP with the blood pump stopped and with the blood circuit and filtrate circuit exposed to the atmosphere. The principle underlying this method of measurement is the same as that of a colloid osmometer (Osmomat 050; Phoenix Science, Inc., Tokyo, Japan).

### Test circuit and measurement method

**Figure 1:**
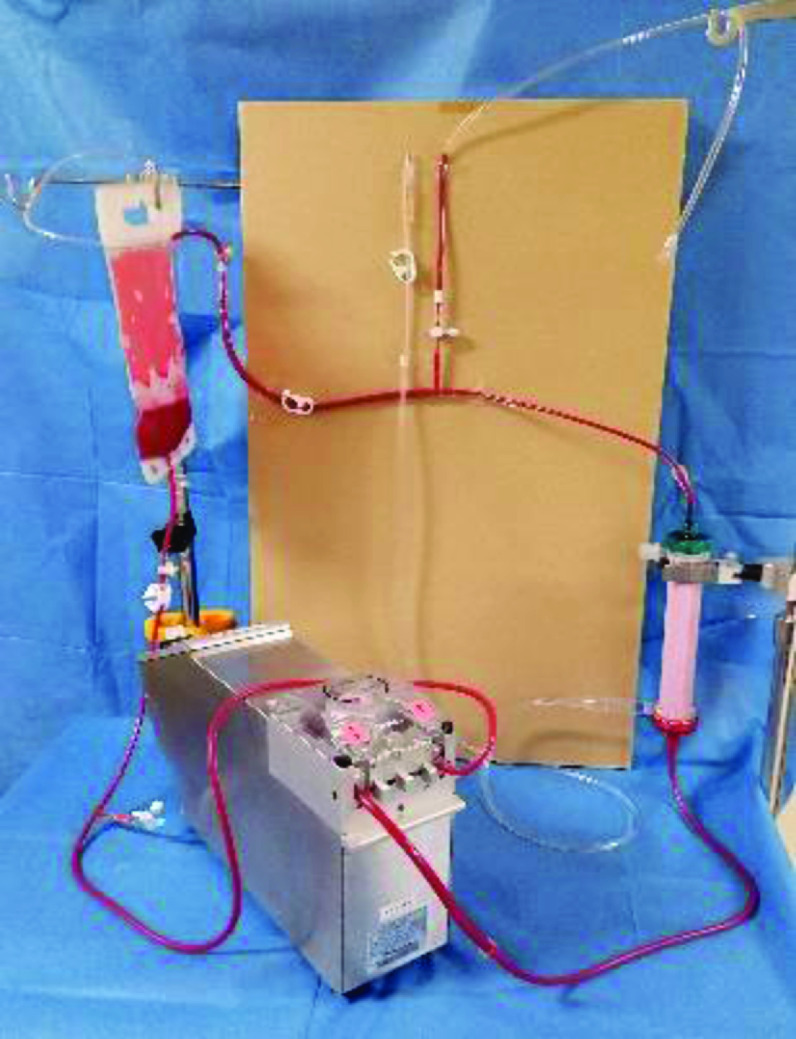
Photograph of test circuit. The test circuit was a closed circuit consisting of an ultrafiltration membrane (BIOCUBE Hemoconcentrator BHC-110; NIPRO Co, Ltd., Osaka, Japan) and a medical soft bag connected by a vinyl chloride tube. The open-air circuit (3.3 mm i.d.) and filtrate circuit (6 mm i.d.) in the blood circuit were fixed vertically side-by-side.

The test circuit was a closed circuit mainly consisting of an ultrafiltration membrane (BIOCUBE Hemoconcentrator BHC-110; NIPRO Co, Ltd., Osaka, Japan) (Table 1) and a medical soft bag connected by a vinyl chloride tube. The test circuit included (1) a roller pump for circulation, (2) a sampling port, (3) a three-way stopcock for switching the blood circuit, (4) an atmospheric release circuit within the blood circuit (inner diameter: 3.3 mm), (5) a filtrate circuit (inner diameter: 6 mm), and (6) a clamp. The atmospheric release circuits in the blood and filtrate circuits were fixed so they were perpendicular to each other (Figure 1). After the roller pump occlusion was adjusted and reflux was confirmed to be absent, the circuit was primed with physiological saline solution and then calcium-chelated bovine blood (Osaka Nanko Zoki Co., Ltd., Osaka, Japan) was added to prepare the test liquid.

**Table 1:** Membrane catalog values used in experiment.

Product name	BIOCUBE Hemoconcentrator BHC-110
Membrane material	Polyethersulfone
Membrane area	1.1 [m^2^]
UFR*	50< [mL/mmHg/h]
Inulin SC**	0.95<
Albumin SC	<0.05
	• UFR measurement conditions: JIS T3250 5.6.3*
	• SC measurement conditions: JIS T3250 5.6.2**
	Blood flow rate = 300 mL/min

*UFR: ultrafiltration rate, **SC: sieving coefficient.

**Figure 2:**
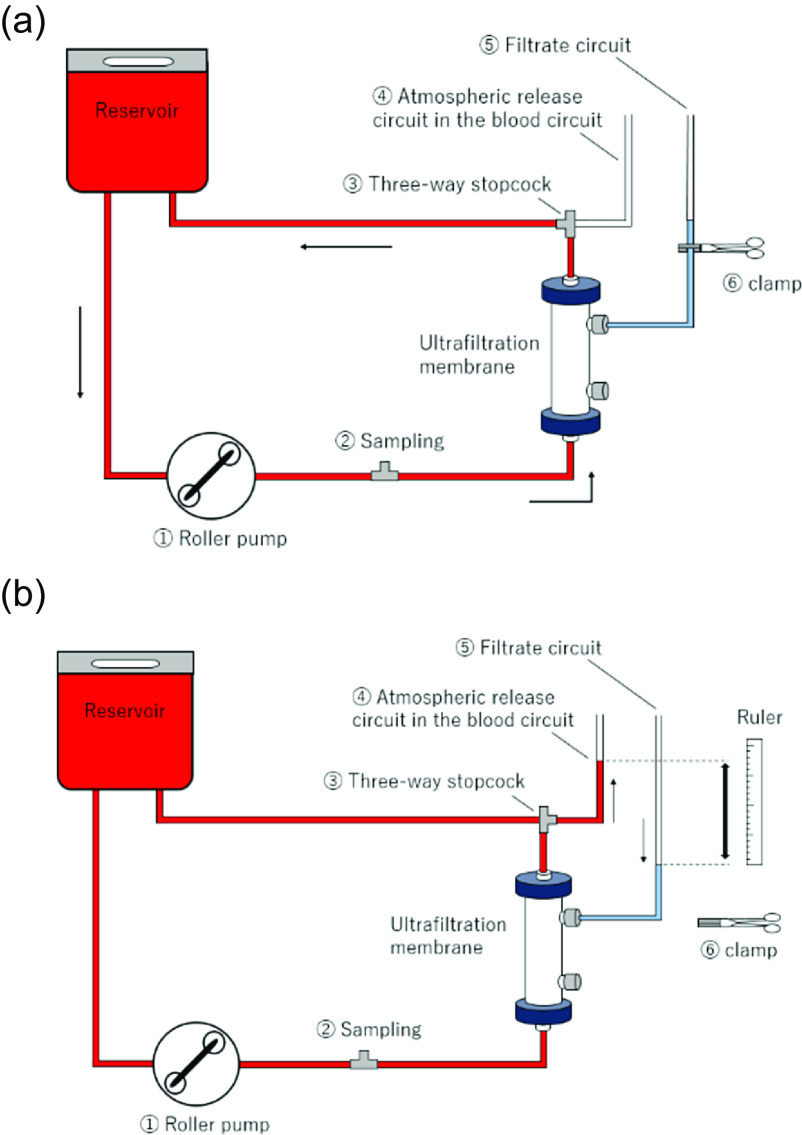
(a) Test circuit diagram (blood circulation before measurement). The atmospheric release circuit of the blood circuit must be emptied. The filtrate level was set at the height of the three-way stopcock for blood circuit switching, the filtration circuit was closed. The blood circuit was cycled for more than 5 min. (b) Test circuit diagram (during colloid osmotic pressure measurement). The roller pump was stopped, the three-way stopcock was switched, and the blood circuit was switched to an open-air circuit. The clamp of the filtrate circuit was then opened to expose the circuit to the atmosphere. After waiting 3 min for the solvent migration in the blood and filtrate circuits to equilibrate, the water level difference created between the two circuits was measured.

In preparation for taking a measurement, the filtrate level was set at the height of the three-way stopcock for blood circuit switching and the filtration circuit was closed. The atmospheric release circuit of the blood circuit must be emptied, which was done by switching the three-way stopcock in the blood circuit (Figure 2b, #3) and returning the blood into the blood circuit via gravity. The blood circuit was cycled for more than 5 min in this state (Figure 2a).

At the time of the measurement, the roller pump was stopped, the three-way stopcock was switched, and the blood circuit was switched to an open-air circuit. The clamp of the filtrate circuit was then opened to expose the circuit to the atmosphere. When both circuits are exposed to the atmosphere and then stopped, the solvent migrates in accordance with the difference in osmolality. The time spent waiting for solvent migration to equilibrate was 3 min (standby time). After the standby time had passed, the water level difference between the two circuits was measured (Figure 2b). The water level difference was measured in centimeters: a 1 cm difference was considered equivalent to a 1 cmH_2_O hydrostatic pressure difference, which was further converted to mmHg by considering 1 cmH_2_O equivalent to 0.74 mmHg (hereinafter referred to as “test method”). To obtain comparative values, measurements were performed using a colloid osmometer to measure simultaneously collected samples. Samples were collected from the sampling port of the circuit (Figure 2b, #2). For re-measurement, the process was repeated from the pre-measurement preparation state.

#### Experiment 1

The bovine blood in the circuit was concentrated using ultrafiltration, and seven concentrations were prepared as test liquids 1–7. The concentration of each test liquid was determined by measuring the hematocrit (Hct) value three times using a blood gas analyzer (ABL800; Radiometer Corporation, Tokyo, Japan), and calculating the mean value.

**Figure 3:**
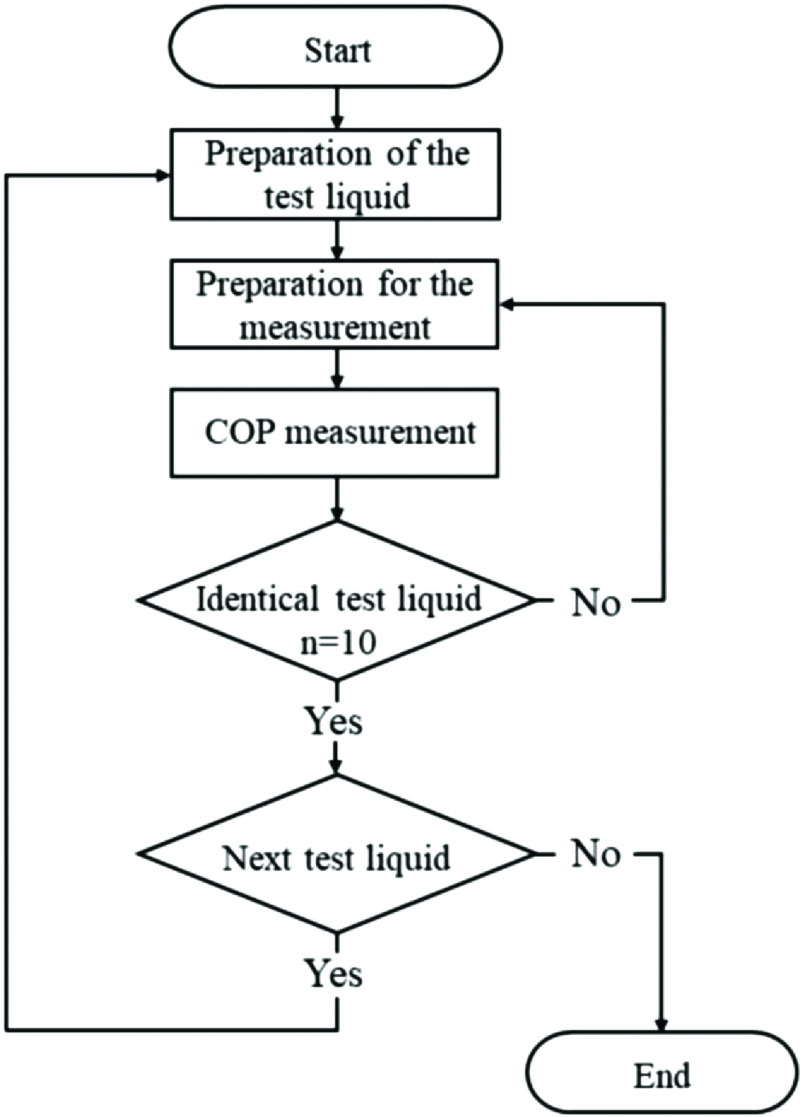
Sequence of events during testing.

For each test liquid, COP was measured 10 times both by the test method and with samples for comparison. For each COP measurement, the circuit was returned to its pre-measurement preparation state, blood was circulated for 5 min, the standby time began, and the next measurement was performed. After the COP of test liquid 1 was measured 10 times with both methods, the blood in the circuit was concentrated to prepare test liquid 2, and the COP was subsequently measured 10 times with both methods. The same procedure was continuously repeated to conduct measurements up to test liquid 7 (Figure 3). Therefore, except for the first test liquid, all test liquids were prepared within the circuit.

To impose a load on the ultrafiltration membrane, ultrafiltration was performed slowly for over 30 min to prepare the test liquid. In addition, the blood flow rate during the preparation of the test liquid was varied. Starting with test liquid 1 (100 mL/min) and test liquid 2 (150 mL/min), the rate was increased by 50 mL/min for each test liquid up to test liquid 7 (400 mL/min).

#### Experiment 2

Three types of test liquid were used (test liquids 8–10). Test liquid 8 was bovine blood, prepared using the same technique as used in Experiment 1, and the concentration was determined based on Hct. Test liquid 9 was prepared by adding 150 mL of aqueous albumin solution to test liquid 8, and then concentrated by removing 150 mL of water using filtration. Test liquid 10 was obtained by adding 100 mL of albumin solution to test liquid 9, removing 100 mL of water by filtration, and concentrating the resulting solution. The aqueous albumin solution was prepared by dissolving albumin powder (FUJIFILM Wako Pure Chemical Corporation, Osaka, Japan) in a physiological saline solution (FUJIFILM Wako Pure Chemical Corporation, Osaka, Japan).

For each test liquid, COP was measured 10 times with the test method and samples for comparison. For each COP measurement, the process was repeated from the pre-measurement preparation state, blood was circulated for 5 min, the standby time began, and the next measurement was performed. After the COP measurement of test liquid 8, test liquid 9 was prepared, and its COP measurement was performed. Similarly, experiments were sequentially conducted to test liquid 10. Therefore, all test liquids were prepared within the circuit. During the preparation of test liquids, ultrafiltration was performed for over 30 minutes and the blood flow rate was varied. The blood flow rates during the preparation of the test liquids were 200 mL/min for test liquid 8, 300 mL/min for test liquid 9, and 400 mL/min for test liquid 10.

### Parameters for evaluation

#### Simultaneous reproducibility

The coefficient of variation (CV) was calculated from the COP values obtained from measuring each test solution 10 times.

#### Correlation

Measurements were plotted on the *x*- and *y*-axes and the Pearson product-moment correlation coefficient was calculated.

#### Measurement time

The time required for measurement was determined as the time required for the completion of solvent migration during the standby time. The standby time was defined as the time required for the completion of solvent migration through osmolality difference and for equilibration; the standby time was set at 3 min after switching the test circuit to measurement status.

### Statistical analysis

Among continuous variables, absolute values are expressed as mean ± standard deviation (*SD*). The Pearson product-moment correlation coefficient was calculated to determine correlations between measurement methods. JMP software (version 14.3.0; SAS Institute, Tokyo, Japan) was used for statistical analysis, and a *p*-value of < 0.05 was considered statistically significant.

## Results

### Experiment 1

The Hct concentrations measured with test liquids 1–7 were listed in Table 2.

**Table 2:** Simultaneous reproducibility with test liquids 1–7 in Experiment 1

	Measurement method (*n* = 10)	Mean COP [mmHg]	*SD*	CV [%]
Test liquid 1 (Hct: 20.2%)	Test method	5.77	3.50 × 10^−2^	0.6
	Colloid osmometer	5.84	4.90 × 10^−2^	0.8
Test liquid 2 (Hct: 22.6%)	Test method	7.09	1.28 × 10^−1^	1.8
	Colloid osmometer	7.08	1.10 × 10^−1^	1.5
Test liquid 3 (Hct: 23.8%)	Test method	8.23	1.49 × 10^−1^	1.8
	Colloid osmometer	8.11	1.00 × 10^−1^	1.3
Test liquid 4 (Hct: 26.0%)	Test method	9.77	8.35 × 10^−2^	0.9
	Colloid osmometer	9.6	7.70 × 10^−2^	0.8
Test liquid 5 (Hct: 28.0%)	Test method	11.65	9.45 × 10^−2^	0.8
	Colloid osmometer	11.48	1.10 × 10^−1^	0.9
Test liquid 6 (Hct: 31.7%)	Test method	15.51	4.07 × 10^−1^	2.6
	Colloid osmometer	14.81	1.80 × 10^−1^	1.2
Test liquid 7 (Hct: 34.8%)	Test method	19.69	3.12 × 10^−1^	1.6
	Colloid osmometer	18.34	6.60 × 10^−2^	0.4

Measurements were performed 10 times for each test liquid and each measurement method, and CVs (%) were calculated. For the COP measurements using the Test Method, all CVs were in the range of 1.44 ± 0.66%. For measurements obtained using the colloid osmometer, all CVs were in the range of 0.99% ± 0.34%. Abbreviations: COP, colloid osmometer; SD, standard deviation; CV, coefficient of variation; Hct, hematocrit value.

#### Simultaneous reproducibility

The CV was within 3% for all 10 measurements of each test liquid. For Experiment 1, the test method was 1.44 ± 0.66% and the colloid osmometer was 0.99 ± 0.34%. Satisfactory results of under 3% were obtained for all cases (Table 2).

#### Correlation

**Figure 4:**
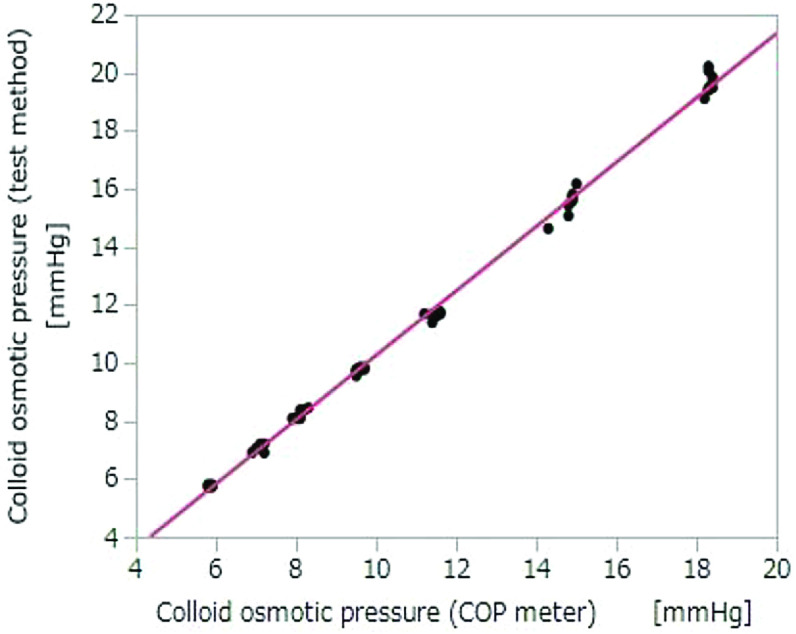
Correlation between COP measurements from the colloid osmometer and by the test method used in Experiment 1. *n* = 70; Pearson’s correlation: *p* < 0.01; *R*^2^ = 0.998. COP, colloid osmotic pressure. *y* = −0.815074 + 1.1085785 × *x.*

COP as measured by the test method and the colloid osmometer demonstrated a strong correlation (Pearson product-moment correlation coefficient *R*^2^ = 0.998, *p* < 0.01). The formula for the regression line is as follows (Figure 4):

(1) y=-0.815074+1.1085785×x.

#### Measurement of time

Although the standby time was 3 min, solvent transfer of test liquid 1 was completed in approximately 10 seconds. Solvent transfer time grew longer as the concentration of the test liquid increased. Solvent transfer for test liquid 7 took approximately 1 min to complete. In all cases, no solvent transfer was observed after 2 min of the 3-min standby time had elapsed.

### Experiment 2

The Hct concentrations measured with test liquids 8–10 were listed in Table 3. The COP of the aqueous albumin solution was 26.8 mmHg using the colloid osmometer.

**Table 3:** Simultaneous reproducibility with test liquids 8–10 in Experiment 2.

	Measurement method (*n* = 10)	Mean COP [mmHg]	*SD*	CV [%]
Test liquid 8 (Hct: 26.2%)	Test method	14.34	3.29 × 10^−1^	2.3
	Colloid osmometer	14.02	1.33 × 10^−1^	0.9
Test liquid 9 (Hct: 25.9%)	Test method	26.36	2.75 × 10^−1^	1.0
	Colloid osmometer	26.46	2.24 × 10^−1^	0.8
Test liquid 10 (Hct: 25.8%)	Test method	33.82	5.68 ×10^−1^	1.7
	Colloid osmometer	33.83	3.10 ×10^−1^	0.9

Measurements were performed 10 times for each test liquid and each measurement method, and CVs (%) were calculated. With COP measurement by the Test Method, all CVs were in the range of 1.67 ± 0.51%. With measurement using the colloid osmometer, all CVs were in the range of 0.90% ± 0.04%. Abbreviations: COP, colloid osmometer; *SD*, standard deviation; CV, coefficient of variation; Hct, hematocrit value.

#### Simultaneous reproducibility

The CV was within 3% for all 10 measurements of each concentration. Overall, for Experiment 2, the test method was 1.67 ± 0.51% and the colloid osmometer was 0.90 ± 0.04%. Satisfactory results of under 3% were obtained in all cases (Table 3).

#### Correlation

**Figure 5:**
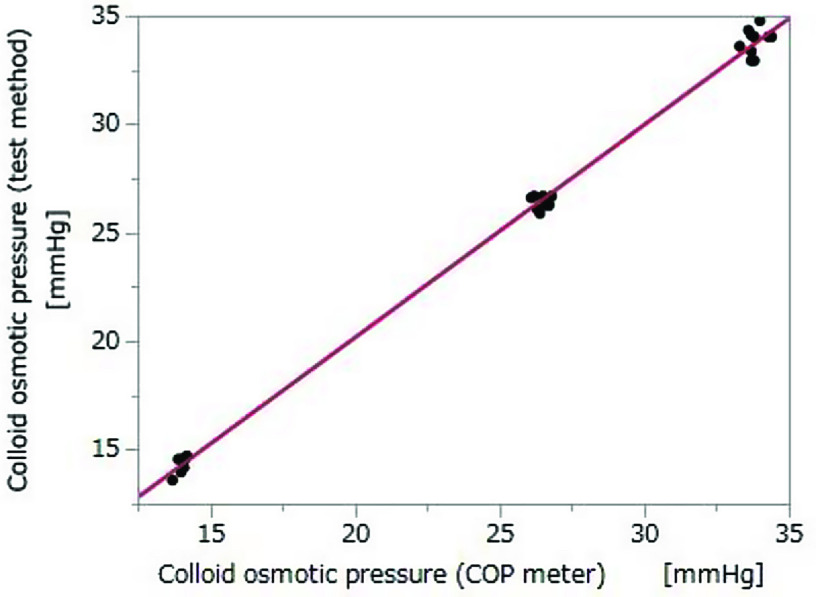
Correlation between COP measurements from the colloid osmometer and by the test method in Experiment 2. *n* = 30; Pearson’s correlation: *p* < 0.01; *R*^2^ = 0.997. COP, colloid osmotic pressure. *y* = 0.5351251 + 0.9813299 × *x*.

COP as measured by the test method and the colloid osmometer demonstrated a strong correlation (Pearson product-moment correlation coefficient *R*^2^ = 0.997; *p* < 0.01). The formula for the regression line is as follows (Figure 5):

(2) y=0.5351251+0.9813299×x.

#### Measurement of time

Although the standby time was 3 min, solvent transfer was completed in under 1 min for test liquid 8. Solvent transfer time increased as the concentration of the test liquid increased. With test liquid 10, moderate solvent migration appeared to occur after 1 min, but no solvent migration could be observed after 2 min had passed.

## Discussion

### Evaluation of measurement accuracy (simultaneous reproducibility)

Simultaneous reproducibility was used to evaluate the accuracy of the measured values. Each measurement was within 3%, which indicates high reproducibility (Tables 2 and 3). The presence of little variability indicates that there may not be many factors contributing to variations in the measurements, even when the person obtaining the measurement changes. This suggests that this measurement method is highly reliable. The absolute value of the colloid osmometer measurement error is ±5 mmHg. It has an error rate of 10% in calculating albumin concentrations [19]. Because the systematic error rate of the test method was unknown, we could not accurately capture all the errors. However, the measurement result was similar to that of the colloid osmometer, which has a measurement error rate of less than 3% CV. Thus, the systemic error rate of the test method is expected to be comparable to that of the colloid osmometer.

Over extended periods of use, the measurement accuracy may be impacted by the performance of the ultrafiltration membranes, which are not constant. The concentration polarization model can explain the filtration performance of semipermeable membranes; substances that cannot pass through the membrane concentrate on the membrane surface increasing transmembrane resistance and decreasing filtration performance. Additionally, fouling is caused by the adhesion of proteins to the surface and interior of the membrane, which similarly increases the membrane passage resistance and reduces the permeability and solute permeability [20–22]. These factors will be affected by the filtered flow rate, blood flow rate, blood concentration, and elapsed time. During our experiment, the ultrafiltration membrane was loaded using variable rates of blood flow. In addition, the blood concentration and elapsed time were varied. However, the test method was not affected by this performance degradation. This may have been due to the fact that the COP measurement was performed after circulating blood for 5 min without filtration in preparation for taking a measurement. This setting was originally devised to avoid measuring COP with concentrated blood immediately after filtration; however, this may have had a cleansing effect by washing out concentration polarization, etc. [20]. Nevertheless, the extent of this effect is unknown. In Experiment 1, once the test liquid was prepared, COP was measured continuously 70 times with a single ultrafiltration membrane. Consequently, the ultrafiltration time with this ultrafiltration membrane lasted over 4 h. A standard cardiopulmonary bypass takes roughly 3 h; longer use of the ultrafiltration membrane did not result in any degradation of the membrane or any other disadvantages that could affect COP measurement. This result implies that measurement remains accurate over long-term use; however, further research is necessary to prove this assumption.

### Correlation with colloid osmometer

The correlation between COP values as measured by the test method and the colloid osmometer was quite high (*R*^2^ = 0.99, Figures 4 and 5). Since the test method and the colloid osmometer operate according to a similar principle and the measurement error is small, we consider this correlation coefficient of 0.99 to be reasonable. Hence, only the measurement error is affecting the correlation. The difference in measurements increases as COP increases; therefore, systematic error may be involved. However, we have not investigated the cause of this error.

Our test method measures the hydrostatic pressure difference at the equilibrium point. The inner diameter of the tube used to measure differences in the fluid level does not affect the hydrostatic pressure. However, if air is mixed into the measurement circuit, there is a possibility that the error will increase. To ensure that the mixed air can be easily removed, a moderately-sized circuit diameter is preferable.

### Measurement of time

Measurements should be easy to conduct and obtain during cardiopulmonary bypass procedures. Our test method does not require zero-point calibration for the colloid osmometer. Complex calculations, such as albumin concentration, are also unnecessary and no sampling is required to conduct the measurement. Hence, considerable time and effort can be saved.

In Experiments 1 and 2, the time required to complete the solvent transfer and reach equilibrium increased with increasing COP. With the colloid osmometer, the time until the results were displayed also increased. This likely results from an increase in the amount of solvent transfer accompanying an increase in COP.

One limitation of our test method is that the amount of time required for solvent transfer to reach equilibrium is unknown. With no apparent signs to confirm the equilibrium point, care must be taken not to measure during solvent transfer. For that reason, we recommend setting a standby time of approximately three minutes. The maximum amount of time required for measurements using a colloid osmometer is four minutes, which does not differ much from the time required for our method.

The effects of measurement time in real-world clinical practice should be considered. Before measurement, blood must be circulated with ultrafiltration stopped, and the concentrated blood must be washed out of the circuit. This exchange of blood takes several minutes; combined with 3 min of standby time, a roughly 5-min period occurs during which ultrafiltration cannot be performed. An inability to perform ultrafiltration for 5 min in cardiopulmonary bypass procedures is unlikely to yield any disadvantages.

### Research applications and limitations

The Hct values of the test liquids used were within the normal Hct range for cardiopulmonary bypass. In addition, the COP of the test liquids included a sufficiently wide range from the normal value of approximately 25 mmHg. As for the load on the filtration performance of the ultrafiltration membrane, the blood flow rate was 100–400 mL/min, and the filtration time was 4 h. Most cardiopulmonary bypass procedures are likely to be within this range. Adding a COP measurement unit to existing ultrafiltration circuits used in cardiopulmonary bypass will make it possible for many facilities to inexpensively conduct measurements during procedures. Specifically, a COP measurement circuit can be added by exposing the blood circuit and the filtration circuit to the atmosphere and arranging them in a perpendicular fashion, incorporating a switchable open-air blood circuit into the blood circuit, and incorporating a switchable open-air filtration circuit into the filtration circuit.

Ultrafiltration membrane selection is important when measuring COP. As measured values are affected by the permeability of the semipermeable membrane, attention must be paid to the sieving coefficient of the membrane albumin. With an ultrafiltration membrane through which albumin passes, COP is also exerted on the filtrate, an action which is predicted to lower measurements. The effects of concentration polarization and fouling due to ultrafiltration have not been fully investigated. In addition, we have not been able to establish a method for cleaning membranes to minimize those effects.

The ends of the filtration circuit and of ultrafiltration circuits in clinical use may be connected to non-sterile containers. In our method, as the filtrate returns to the blood chamber, sterility in the filtration circuit must be maintained. We have not solved this problem with our experimental circuit. For clinical applications, it will be necessary to devise further circuits, such as a filtrate switching circuit.

COP measurement using an ultrafiltration membrane solves the problems posed by existing COP measurement methods. It is a simple, inexpensive measurement method that can be used widely in clinical practice. Although the measurement circuit requires further improvements, we expect clinical applications to emerge in the future.

In conclusion, the COP measurement with the test method using an ultrafiltration membrane revealed a strong correlation with the results of colloid osmometers currently in use, and the test method may be a useful technique for simplified COP measurement during cardiopulmonary bypass. This study was performed *in vitro*; by conducting *in vivo* experiments, the validity of the measurements can be established. This will provide appropriate COP control values for extracorporeal circulation.

## Data Availability

The research data associated with this article are included within the article.
